# Brain cancer and pesticide relationship in orchard farmers of Kashmir

**DOI:** 10.4103/0019-5278.75694

**Published:** 2010

**Authors:** Abdul Rashid Bhat, Muhammed Afzal Wani, A. R. Kirmani

**Affiliations:** Department of Neurosurgery, Sher-i-Kashmir Institute of Medical Sciences, Srinagar, Kashmir, India

**Keywords:** Brain cancer, Kashmir, orchard farmers, pesticides

## Abstract

**Background:**

The increasing trend in the incidence of primary malignant brain tumors in orchard farmers and their families in Kashmir.

**Aim:**

To determine the relationship between the patients of primary malignant brain tumors and their occupation.

**Materials and Methods:**

Retrospectively, case files along with death certificates of 432 patients of primary malignant brain tumors and 457 controls (non-tumor neurologic diseases), admitted for treatment simultaneously over a period of 4 years from January 2005 to December 2008, to the Neurosurgery, Sher-i-Kashmir Institute of Medical Sciences (SKIMS), Kashmir, were studied. Follow-up and family interaction was established.

**Results:**

Analysis revealed that 90.04% (389 out of 432) patients were orchard farm workers, orchard residents and orchard playing children exposed to the high levels of multiple types of neurotoxic and carcinogenic (chlorpyriphos, dimethoate, mancozeb and captan) chemicals for more than 10 years [relative risk (RR) = 10.6; odds ratio (OR) = >10; 95% confidence interval (CI) = >25-40]. The 9.96% (43 out of 432) patients were not exposed to pesticides. On the other hand, only 19 patients out of 457 controls had recorded history of pesticide exposure and 438 were unrelated to pesticides. Out of 389 patients, 71.7% (279 out of 389) were males and 28.3% (110 out of 389), including six members of three families, were females (one male child).

**Conclusion:**

All orchard-related 389 patients had high-grade tumors as compared to the non-pesticide tumors. Mortality in pesticide-exposed tumors was 12%. The higher or upper-normal levels of serum cholinesterase (AChE) were observed in 54.7% (213 out of 389) patients and decreased levels were found in only 45.3% (176 out of 389) orchard-related patients (RR = 19.4; OR = >5; 95% CI = >1-10). Although serum AChE levels were a routine investigation in malignant brain tumors, this was not a routine in other neurological conditions (hospitalized controls). The familial gliomas have shown an emerging trend in the orchard residents of valley of Kashmir.

## INTRODUCTION

Occupational health hazards are well known. The widespread use of pesticides in the agricultural industry to control the insects, pests and fungus and to enhance the crop and fruit production is recognized as a major chemical health hazard for the orchard workers, residents and children by direct contact and by polluting the aerial, soil and water environment. The residual concentrations of these toxic chemicals in the farm workers have led to a variety of neurological dysfunctions.[1–3] Because of the similarity in the brain biochemistry, the pesticides are particularly neurotoxic to the humans, and the most lethal of them are organophosphates, carbamates and ethylenebisdithiocarbamates (EBDCs).[4] The primary action of the organophosphates and carbamates is to irreversibly inhibit the activity of the enzyme acetylcholinesterase (AChE) that hydrolyzes the neurotransmitter acetylcholine in both the peripheral and central nervous systems. This causes accumulation of acetylcholine at cholinergic synapses, leading to over-stimulation of muscarinic and nicotinic receptors, and thus, neurotoxicity.[5] Occupational exposure of workers in different industries like rubber, oil refinery, chemical plant and polyvinyl chloride have been reported to have elevated the risk of developing brain tumors.[6] The etiologic importance of exposure to pesticides has been reported by case-control studies on childhood brain tumors arising after exposure to chlordane and heptachlor.[7,8] This was confirmed by a report on patients who died from malignant tumors, among whom a high level of organochlorine compounds was found in the adipose tissue of those who had glioblastomas.[9] A study reported two of seven patients from a cluster of primary brain tumors, who were exposed to the pesticides.[10] Pesticides are suspected to be the potent risk factors for the lethal brain tumors, especially gliomas, in the children and adults.[11] Annual European Union (EU) pesticide use includes 0.108 million tons of fungicides, 0.08 million tons of herbicides, 21,000 tons of insecticides and 7000 tons of growth regulators, amounting roughly to half a kilogram of active substances for every man, woman and child living within the EU.[12] The fruit production of Kashmir province of JandK, India, is 1.5 million metric tons annually from a total orchard area of 0.2 million hectares, which is sprayed and fogged with 7750 metric tons of fungicides and 3186 metric tons of insecticides, right from March to November every year, in 10 recommended scheduled stages from green tip and pink budding of the trees (pre-bloom) up to and after the harvest of the fruits (post-harvest), though unofficial frequency of unscheduled sprays by farmers is increased to 15–20. The excessive use of synthetic pesticides for the last three decades and increase in admission of high-grade malignant brain tumors, with history of pesticide exposure, to the Neurosurgical Centre, Sher-i-Kashmir Institute of Medical Sciences (SKIMS), Kashmir, in the last 10 years has elicited high degree of suspicion of a link between the pesticides and malignant brain tumors (brain cancer).

## MATERIALS AND METHODS

The Department of Neurosurgery, SKIMS, Srinagar, Kashmir province of JandK, India, caters to about 7–8 million ethnic non-migratory population of Kashmir province as a single center. The case files, including death summaries, of 432 patients admitted from January 2005 to December 2008 (4 years) and proved histopathologically as primary malignant brain tumors, were studied. The controls, 457 patient files with non-tumor brain conditions like brain abscesses, tuberculomas, meningitis, strokes, multiple sclerosis, etc., admitted during the same period were also studied. All metastatic lesions to brain were excluded. The historical (direct occupational exposure: farm-workers and indirectly: residents and playing children), clinical, biochemical, radiological and histopathological findings were recorded. The attendants as well as patients named and identified the used, open and sealed chemical (pesticide) containers and packs to which the patients and the controls were exposed. The patients and the families were contacted to collect further information for follow-up and to select randomly the familial controls. The blood samples were collected from 171 family controls and 180 more controls selected from general population randomly after age, sex and socioeconomic matching with the patients. This made a total of 808 controls whose serum AChE was estimated. The historical information collected was age, sex, socioeconomic status, form of exposure, e.g., mixer–loader applicators, sprayer, fogger in the case of adults, number and type of chemicals exposed to, years of exposure and lifelong jobs, orchard guards and supervisors, whether following pesticide applicator precautions like avoiding use of expired drugs or spurious drugs, use of hand gloves, hand wash, masks and goggles, head gear and uniform, eating unwashed fruits from the orchards, location of residential house, location of drinking water source, location of play grounds of children and frequency of female family members visiting and working in the orchards. The serum AChE levels of patients (the levels have been recorded in the file sheets of brain malignancy only, not in hospitalized controls) were recorded and the levels were checked in the family and general controls. The patients and controls with carcinomas, metastasis, hepatitis, acute infection, cirrhosis, nephrotic syndrome, thyrotoxicosis, hemochromatosis, muscular dystrophies and psychiatric disorders were excluded. The data collected were compiled, analyzed by analysis of variance (ANOVA) using the SPSS, version 11.5, statistical program and unconditional logistic regression was used to compute odds ratio (OR) and 95% confidence interval (CI), adjusted for the matching variable (age, sex, orchard workers, non-pesticide–exposed cases, cholinesterase). The law of variance was applied wherever required.

## RESULTS

The highest number of cases was from the Baramulla, Anantnag and Budgam districts [Table 1] and the most hospitalized controls were from the Srinagar district. The family and general population controls were equally selected from all the orchard districts.

**Table 1:** Kashmir orchard areas, number of pesticide-exposed cases and approximate pesticide consumption

Orchard district	Orchard area (ha)	Pesticides utilized (MT)			No. of cases
		Chlorpyriphos	Mancozeb	Captan	
Budgam	29,572	Pink-bud stage 50% 3 l/ha Fruitlet stage 50% 4 l/ha	Fruitlet stage 30% 12 kg/ha Pre-harvest 30% stage 12 kg/ha	Fruitlet stage 10% 12 kg/ha Pre-harvest stage 20% 12 kg/ha	55
Anantnag	28,697				63
Barmaulla (Varmul)	28,031				88
Kupawara	25,583				45
Shopian	24,073				50
Kulgam	18,926				34
Pulwama	17,664				25
Others (Srinagar, etc.)	20,563				29
Total	193,109	3186 MT	3400 MT	4350 MT	389

Chlorpyriphos, mancozeb and captan are European Union (EU) labeled carcinogens

The age and sex of patients showed that adult males had outnumbered others. The 389 (90%) cases out of 432 primary malignant brain tumors (proved by histopathology after open or closed biopsy, metastasis, excluded) had exposure to pesticides in various ways while 43 had no exposure. Among 457 controls, only 19 had pesticide exposure. Thus, the OR of more than 10.00, 95% CI of more than 25–40 and a relative risk (RR) of 10.6 are significant. Out of 389 (100%) patients, there were 31 (7.9%) children, 304 (78.1%) adults and 54 (13.9%) elderly people [Table 2]. A total of 279 (71.7%) males and 110 (28.3%) female patients were exposed to pesticides. The eldest patient was a 75-year-old male with a hemispheric glioblastoma multiforme and the youngest was an infant female baby with medulloblastoma. A mortality of 12% (47 cases out of 389) was revealed among orchard (pesticide-exposed) farm workers as compared to 7% (3 out of 43) deaths in non-pesticide workers.

**Table 2:** Age and sex of 389 cases

Age span	Total no. of cases	Males	Females
Children (<18 years)	31	21	10
Adults (19–50 years)	304	207	97
Elderly (51–80 years)	54	51	3
Total	389	279	110

The ways of exposure were different. The 207 males out of 304 adults (age 19–50 years) were mostly pesticide mixers, sprayers, foggers and orchard tillers. The 97 adult females were frequent visitors and part-time orchard workers. Of these, 23 pregnant females had been exposed to the pesticides in their antenatal and postnatal periods and 11 were lactating mothers. Most adults had more than 10–30 years working history in different (apple, walnut, almond, cherry, pear, grapes, peach, apricot, etc.) orchards. All the 54 elderly (age 51–80 years) patients were males except 3 females and all were lifelong orchard workers, mostly weed handlers, fillers and orchard supervisors [Table 2]. The orchard area with most cases (88 patients out of 389 cases) was Sopore, Baramulla (Varmul) and 63 cases from Anantnag, followed by Budgam, Shopian, Kupwara, Srinagar, etc. The orchard area of these districts amounts to about 140,000 ha of a total of 0.193 million hectares, with an annual consumption of thousands of metric tonnes of pesticides when calculated at the officially recommended doses. Many of these are carcinogens, e.g., chlorpyriphos, captan, mancozeb, etc. [Table 1]. There were 81 orchard residential families; 85 members of them suffered malignant brain tumors with 6 females (2 adults and 4 children) and one male child from three families, i.e., mother/daughter, three sisters and two siblings (sister/brother). The 31 children either lived in the residential houses with their parents or had exposure by spending most of the time schooling and playing in and around orchards. The drinking water from the pesticide contaminated orchard wells was the source of pesticide exposure for all ages and sexes. Out of 432 cases, 43 (non-pesticide) primary malignant brain tumors were not associated in any way with orchards or pesticides. From 457 hospital controls, only 19 controls had history of pesticide exposure and 438 had no relation to pesticides. The RR for those exposed to pesticides and having primary malignant brain tumors is 10.66. Residential houses constructed in the orchards are at risk of pesticide contamination due to unwashed footwear of farm workers, farming tools, vegetables, fruits, hay stacks for cattle carried into homes and contaminated orchard dust carried by the winds. The drinking water wells in the orchards and orchard residential houses are contaminated due to the direct spill of the chemical mixture into the wells while constituting the spray and indirectly by the rain washings into the soil. Thus, drinking water site is a persistent source of pesticide exposure.

Among the symptoms and signs, the most common presenting symptom was headache, followed by epilepsy, vomiting and the visual blurring. The most common sign found was papilloedema. The computed tomography (CT) scan and magnetic resonance imaging (MRI) brain were the diagnostic tools of choice. All patients were operated upon and histological diagnosis was sought and recorded.

**Figure 1:**
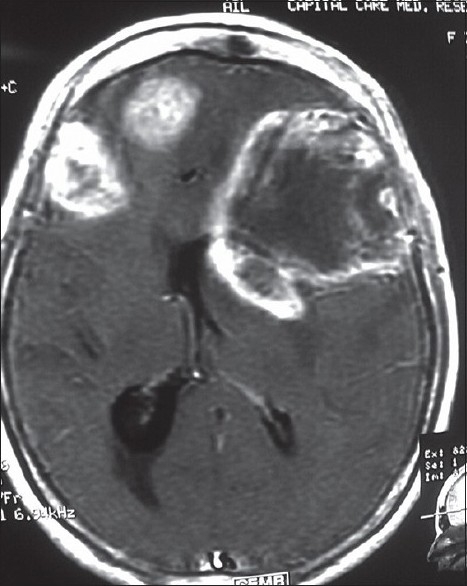
T1WI axial MRI brain of an orchard worker showing multicentric glioma

**Figure 2:**
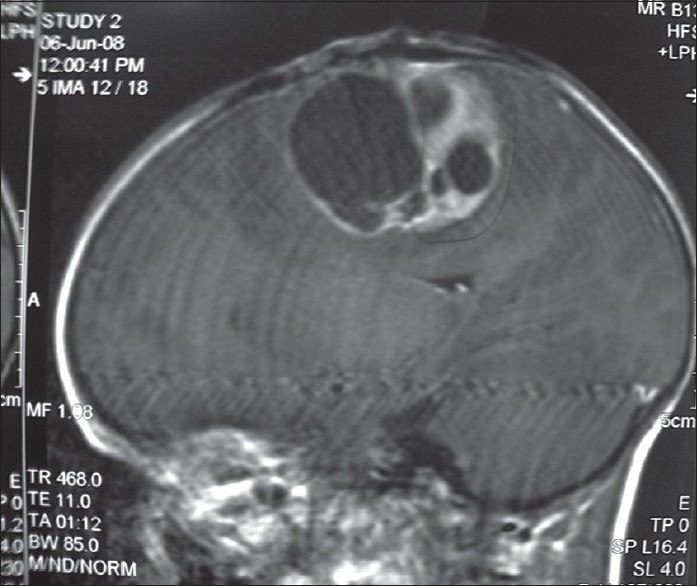
Saggital MRI brain with a glioblastoma multiforme in a male farm worker

When histological types of malignant brain tumors were analyzed, it was found that most of the 389 orchard workers with malignant brain tumors were having highly malignant astrocytomas, glioblastoma multiforme, anaplastic oligodendrogliomas, ependymomas, choroid plexus papillomas, medulloblastoma, etc., as compared to the 43 non-pesticide malignant brain tumors [Table 3]. The children had mostly primitive neuroectodermal tumors. Some of the patients had high-grade multicentric type of gliomas with worst prognosis [Figures 1 and 2].

**Table 3:** Histological types of primary malignant brain tumors in 432 cases of orchard workers and non-pesticide cases

Histological type	No. of orchard workers	No. of nonpesticide cases
Glioblastoma multiforme*	96	5
Anaplastic astrocytomas (WHO grade III)	67	4
Astrocytoma (WHO grade II)	38	15
Anaplastic oligodendroglioma (WHO grade III)	28	2
Oligodendroglioma (WHO grade II)	30	11
Anaplastic ependymoma (WHO grade III)	21	-
Ependymoma (WHO grade II)	28	6
Anaplastic oligo-astrocytoma (WHO grade III)	7	—
Mixed oligo-astrocytoma (WHO grade II)	10	—
Gliosarcoma	11	—
Gliomatosis cerebri	6	—
Choroid plexus papilloma	19	—
Ganglioglioma	5	—
Esthesio neuroblastoma	2	—
Pineocytoma	3	—
Medulloblastoma*	15	—
Retinoblastoma	3	—
Total	389	43

**Figure 3:**
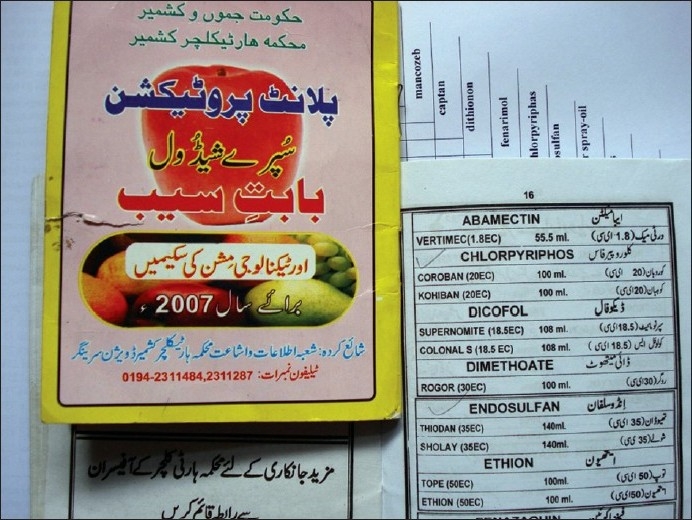
Violation of officially recommended spray schedules in the orchard farms of Kashmir is rampant

**Figure 4:**
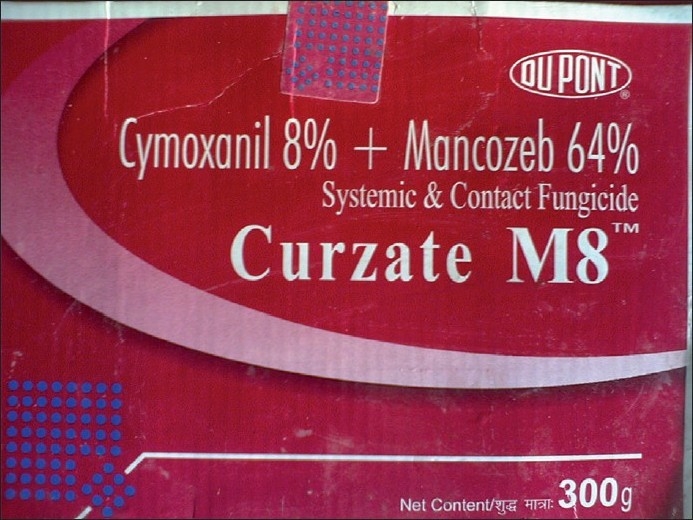
The fungicide mancozeb (ethylenebisdithiocarbamate), a carcinogen, has been long in use in all orchards of Kashmir

**Figure 5:**
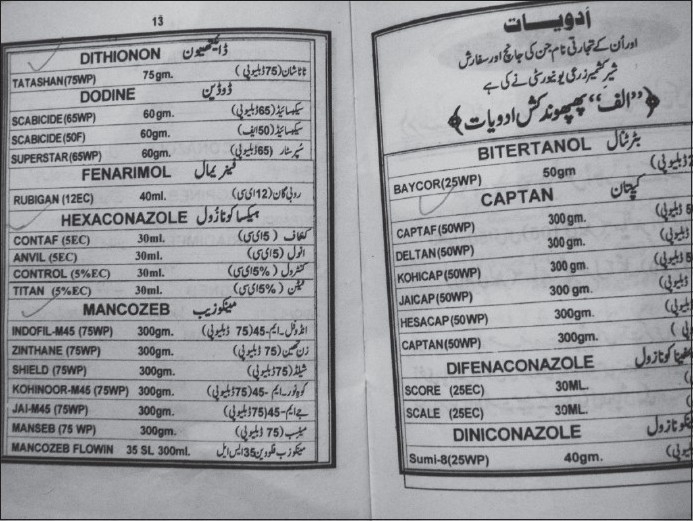
All types of pesticides, irrespective of their health hazardous activity, are used by the Kashmiri farmers

**Figure 6:**
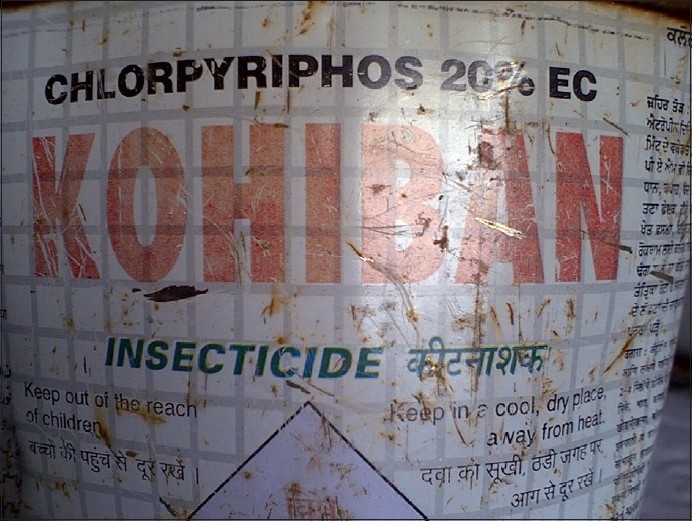
Chlorpyriphos, the most studied organophosphate, is known to act through non-cholinergic mechanisms to induce brain cancer

**Figure 7:**
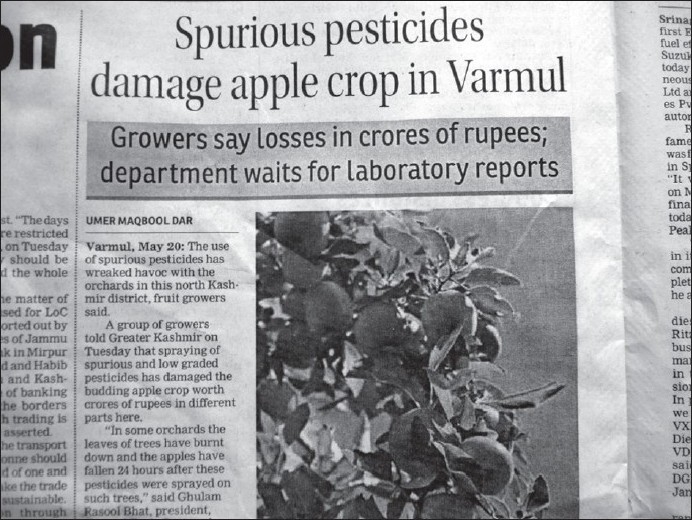
The low-quality, unsampled, spurious pesticides— An ominous human health hazard

There were varied pesticides and AchE level correlations. The number of pesticides used by the orchard farmers in Kashmir was more than 30 fungicides, insecticides, acaricides, etc., most of these spurious and sold without licenses. Among these, chemical groups like organophosphates, organochlorines, carbamates, EBDCs, pyrethroids, phosphines, dicarboximides, inorganics, ureas, dinitroanilines, etc., are still used on a large scale. The officially recommended dithiocarbamate fungicide and EU labeled carcinogen, mancozeb, has been sprayed over in the apple orchards at a dose of 12.00 kg/ha twice in a season (Plant Protection Spray Schedule, Information Office, Department of Horticulture Kashmir Division, Srinagar) [Figure 3] in fruitlet stage (pen size) and pre-harvest stage, which alone amounts to about 700 MT (metric tonnes) per season [Table 1]. Mancozeb is in use for the last 20 years in the Kashmir valley [Figure 4]. Meanwhile, this fungicide is much abused unofficially by the farmers, by its excessive use on the apple trees in the stages not recommended and on un-recommended types of fruits like walnut, almond, cherry, etc. [Figure 5]. Similarly, the use of captan, a dicarboximide fungicide and EU labeled carcinogen, used excessively by the orchard farmers than the recommended doses of 12.00 kg/ha, is directly absorbed through skin, inhalation and ingestion. This was extensively sprayed in all stages and seasons of fruit growth by the farmers without wearing any special body gear or uniform, bare handed with short applicators [Figure 5]. Among the organophosphates, the most used chemicals were chlorpyriphos (dose 50% 4 l/ha) and dimethoate [Figure 6]. Both the neurotoxic insecticides depress the serum AChE levels [Table 3 and 4]. However, results revealed that only 45.3% (176 out of 389) patients of those exposed to pesticides over more than 10 years had lower levels (<3167 u/l) of serum AChE. This also revealed normal AChE (3167–6333 u/l) in 22.8% (89 out of 389) patients and higher levels of >6334 u/l in 31.9% (124 out of 389) patients, equally in both the sexes [Table 4]. But the calculated RR of having lower AchE against upper normal and higher levels together for those exposed to pesticides is 19.4. This was also proved by an OR of >5.00 and 95% CI of >1–10. However, no recorded levels of AChE in the case of hospitalized controls were found. All the family and general population controls had either higher levels of serum AChE or normal levels. Among organochlorines, endosulfan has been the choice of farmers, which is a known convulsant, mutant and carcinogen. This is used on all trees in almost every stage of fruit growth [Figure 3]. The farmers have used cheaper and spurious drugs in greater quantities, sold without licenses, than quality and officially sampled drugs [Figure 7]. The instructions labeled on the pesticide packs, including date of expiry, were neither attended to nor followed by the farmers.

**Table 4:** Serum cholinesterase (AChE) levels in pesticide-exposed and non-pesticide–exposed cases and controls

Cases/control Cases	Serum cholinesterase (AChE) levels (u/l)			Total
	Decreased (<3167)	Normal (3167–6333)	Increased (>6333)	
Pesticide-exposed	176	89	124	389
Non-pesticide exposed	1	38	4	43
Subtotal	177	127	128	432
Hospital control				
Pesticide-exposed	2	10	7	19
Non-pesticide exposed	17	354	67	438
Subtotal	19	364	74	457
Family control				
Pesticide-exposed	15	68	23	106
Non-pesticide exposed	5	23	37	65
Subtotal	20	91	60	171
Generation control				
Pesticide-exposed	3	13	9	25
Non-pesticide exposed	12	84	59	155
Subtotal	15	97	68	180
Grand total	231	679	330	1240

*P* value = 0.000001

**Table 5:** Retrospective case–control studies which evaluated the pesticide–brain tumor link

Study	No. and source of cases	No. and source of controls	Type of exposure	Method	Results
Thomas *et al*.[6]	718 brain tumor deaths	738 controls	Occupation	Death certificates	OR = 0.8; 95%
					CI = 0.4–1.8 (NO)*
Speers *et al*.[36]	202 Texas males died of gliomas	238 males	Occupation	Death certificates	OR = 0.61; 95%
					CI = 0.3–1.22 (NO)
Musicco *et al*.[27]	420 patients of gliomas hospitalized	465 non-glioma brain tumors and 277 non-tumor patients of neurologic disorders	Occupation and residence	Interview	RR = 1.6; 95%
					CI = 1.06–2.42 (SIG)*
Reif *et al*.[37]	452 registered brain cancer patients	19,452 non-brain cancer patients	Occupation	Interview	OR = 1.3; 95%
					CI = 1.0–1.7 (SIG)
Schlehofer *et al*.[38]	226 patients with primary brain tumors in Germany	418 population controls	Occupation	Questionnaire	RR = 1.1; 95%
					CI = 0.7–1.9 (NO)
Forastiere *et al*.[39]	1674 male cancer deaths from Italian agricultural region	Random samples of 480 individuals selected from same regional mortality file as being deceased from all causes		Regional mortality file (death certificates)	OR = 1.04; 95%
					CI = 0.43–2.44 (NO)
Rashid *et al*, Kashmir study	432 patients of gliomas hospitalized	457 hospital controls, 171 family and 180 general controls	Occupation and residence	Hospital files, medical records and family/patient interaction	Cases: RR = 10.66; OR = >10.0; 95%
					CI = >25 to >40 AChE: RR = 19.4; OR = >5; and 95%
					CI = >1–10

* SIG: Significant, NO: Not significant

Familial Gliomas were found. Three families among 81 orchard residential families had (seven members i.e. six females and one male child) more than one member with primary malignant brain tumor. The first belonged to Baramulla (Varmul) and the two members were mother with anaplastic astrocytoma and the 10-year-old daughter with a medulloblastoma. The second family comprised three sisters from the Srinagar district (least in orchard area), who presented from eldest to youngest in a span of 3 years. The eldest sister had ependymoma, the younger medulloblastoma and the youngest had choroid plexus papilloma. The third family from Baramulla had two siblings, i.e 12 year old sister with oligodendroglioma and 10 year old brother with fourth ventricle ependymoma. The reason for familial primary malignant brain tumors could be common stimulatory agent. These two families revealed extensive use of multiple pesticides including mancozeb, chlorpyriphos and captan. The history revealed that about 16 female patients of primary brain cancer had multiple abortions, still births and babies delivered with congenital anomalies of brain and spinal cord.

## DISCUSSION

As a background, the major economic source of the Kashmir province of India is the production of various fresh and dry fruits, which are spread over an area of around 0.2 million hectares, of which 0.11 million hectares (>50%) are under apple production, involving about 40% population of the Kashmir directly (farmers, chemical sprayers, etc.) and indirectly (children playing in and around orchards, residential houses in orchards). Millions of tons of pesticides, insecticides and fungicides (chemicals like chlorpyriphos, mancozeb, captan, dimethoate, phosalone, etc.) are being used by the orchard farmers to spray the plants, fruits and the leaves at different stages of growth to avoid the infestations and destruction of the fruits. For the last two decades, the farmers have favored and adapted to the newer synthetic but hazardous fungicides and pesticides, never applied before, to enhance the fruit production by replacing the older, relatively non-hazardous inorganic sulfur [Table 1]. The incidence of the malignant brain tumors in Kashmir has shown reportedly an upward surge in the last 10 years, especially in the elderly and orchard farming districts. A study showed glioblastomas multiforme accounting for 69.4% of all gliomas.[13] Headache and epilepsy have been the most common symptoms and signs as reported in a study.[14] This finding is similar to that of this hospital-based case–control study. Epidemiological studies show associations with neurodevelopmental deficits on exposure to the mixed pesticides. Laboratory experimental studies using model compounds suggest that many pesticides currently used in Europe – including organophosphates, carbamates, pyrethroids, EBDCs and chlorophenoxy herbicides – can cause neurodevelopmental toxicity.[4] The organochlorine pesticide, endosulfan, is reported to be lipophilic in nature and neurotoxic.[15] Endosulfan is a epileptogenic, and experimentally, a teratogenic, tumorogenic and a carcinogenic. The human mutation is reported.[16] The present study on the orchard farmers of Kashmir revealed 31 children with brain cancer, the youngest of these being an infant. The prenatal, natal and postnatal exposure to pesticides has created enough of doubt concerning the toxicity and mitotic abnormalities in the developing brain of pregnant patients. The 16 female patients had delivered babies with congenital central nervous system malformations. Endosulfan has been one of the most used and abused pesticides. Chlorpyriphos is the most extensively studied organophosphate with respect to the neurotoxicity in the laboratory models where prenatal and neonatal exposure has led to a variety of behavioral abnormalities in both the mice and rats. Chlorpyriphos exposure in rat embryo cultures at concentrations comparable to those found in human meconium showed mitotic abnormalities and apoptosis during the neural tube development stage. However, exposure during gestation led to deficits in brain cell numbers, neuritic projections and synaptic communication, which emerged in adolescence and continued into adulthood. The deficits elicited by prenatal exposure to chlorpyriphos are evident even at exposures below the threshold for detectable AChE inhibition, i.e., far below the 70% inhibition of AChE required for systemic toxicity in adults. These findings suggest that chlorpyriphos also acts via non-AChE inhibition mechanisms to cause neurotoxicity.[17–20] The Kashmir study shows difference in the levels of AChE among the patients who are pesticide-exposed farm workers [Table 4]. The non-cholinergic mechanisms of chlorpyriphos are not clear but a possible target may be the signaling cascades involved in neuronal and hormonal inputs, including the cyclicAMP (cAMP)-protein kinase A cascade, receptor signaling through protein kinase C, and direct effects on the expression and function of nuclear transcription factors mediating the switch from proliferation to differentiation, including c-fos, p53, AP-1, SP 1 and CREB (Ca^2+^/cAMP response element binding protein).[21] This has opened the possibility that organophosphates may have compound specific effects unrelated to the common AChE inhibition, as shown by the similar effects of two organophosphates, chlorpyriphos and diazinon, on the gene expression of neonatal rat brain with the doses not inducing biologically significant AChE inhibition and yet both have notable disparities.[22–24] The pesticide-exposed orchard farmers of Kashmir with primary brain cancer showed a lot of variation in the levels of serum AChE. Although 45.3% patients had depressed levels of AChE, 54.7% had normal and higher serum AChE levels, but an OR of more than 5.00 and 95% CI of more than 1-10.0 and an RR of 19.4 predicts decrease in AChE levels more often [Tables 4 and 5]. The non-cholinergic mechanism, slow and chronic poisoning with chlorpyriphos and mixed exposure to pesticides may be the probable causes for the neurotoxicity and stimulation of brain cancers in Kashmir. The manufacturer of chlorpyriphos, DowElanco, has agreed to restrict its recommended uses in fleas, ticks and pets.[25] A study that assessed mortality rates among vineyard workers in 89 geographical locations in France found a significantly higher incidence of brain cancer among those exposed to pesticides compared to the French population.[26] Similar to this study, the Kashmir study reveals the highest incidence of primary brain cancer in the geographical areas of Baramulla (Sopore, Varmul), Anantnag, Budgam, Shopian and Kupwara, which comprise most of the orchard areas of Kashmir [Table 1]. A total mortality of 12% was recorded in the pesticide-exposed orchard farmers as compared to 7% non-pesticide patients. Many farmers using fungicides reported the use of commercial compounds of copper sulfate, some of which contain methylurea, a carcinogen of nervous system in animals.[27] In Europe, the grapes receive 15% of total synthetic (active substance) pesticides applied to major crops. The synthetic fungicides applied to grapes include substances like dithiocarbamates, a family of chemicals in which pesticides like maneb and mancozeb are EU classified carcinogens. Among the hazardous pesticides commonly found in the food items purchased in EU are proven carcinogens like maneb, procymidone, iprodione and captan. While procymidone has 93% and iprodione 100% transfer rate from grapes to wine, both are proven carcinogens as reported by French Ministry of Agriculture.[28,29] Compared to Europe, the Kashmir province of J and K state, India, is 1/20^th^ in area. The amount of pesticides and fungicides sprayed amounts to thousands of metric tonnes of mancozeb, captan, chlorpyriphos, dimethoate, etc. Familial gliomas have been reported in many studies but not in pesticide workers. There are many reports where brothers, parent and child in the families suffered similar types of brain cancer.[30,31] The present study recorded two families with five female members having deadly primary brain cancer and some of the cases even with multicentric high-grade gliomas. Dithiocarbamates are non-cholinesterase inhibiting and sulfur-containing carbamates which are primarily used as fungicides and herbicides. There are four major classes, of which the EBDCs like mancozeb, maneb and zineb are EU labeled carcinogens. Mancozeb is linked to the uncoupling of the mitochondrial electron transport chain which generates reactive oxygen species leading to neuronal toxicity.[32] Owing to the rapid dermal, inhalational and oral absorption of mancozeb, the un-gloved, un-masked and un-protectively clothed Kashmiri orchard workers who spray tonnes of this pesticide are much vulnerable to its toxicity and carcinogenic effects [Table 1]. Epidemiologically, it is difficult to study the risk of a specific pesticide as a cause of brain tumor because the exposure is not limited to one chemical only but a mixture of multiple pesticides in a spray or a fog.[27] A case–control study revealed that among household pesticides, pest-strips have been reported to be the most consistent pesticides related to a variety of childhood cancers including brain cancer.[33] The childhood cancers reported in the pesticide workers of Kashmir study was 7.9% (31 out of 389) and most of these were primitive neuroectodermal tumors which have worst prognosis and fatal outcome [Table 2]. However, authors in an epidemiological review revealed that great majority of cohort studies of chemical workers employed in the manufacture of pesticides did not indicate an excess of brain cancer mortality. But few cohort studies of pesticide applicators showed elevated RR for excess mortality due to brain cancer.[34] The present Kashmir study finds substantial amount of evidence in favor of a relationship between the malignant brain tumors (brain cancer) and pesticide workers in the orchard farms of Kashmir, with a significant RR (relative risk) of 10.66 and OR of more than 10.0 and 95% CI of more than 25–40 [Table 4 and 5]. The raised RRs, ORs and CIs are significant and is due to the pesticide-exposed orchard farmers outnumbering the non-pesticide cases and pesticide-exposed controls. Evaluation of a series of retrospective case–control studies revealed significant link between occupation and the brain cancer. The studies conducted by Musicco *et al*., in 1988, showed a significant RR of 1.6 and 95% CI of 1.06–2.42, and Reif *et al*., in 1989, reported a significant OR of 1.3 and a 95% CI of 1.0–1.7. However, Thomas *et al*., and others depicted non-significant relationship between the two.[27,35–39]

## CONCLUSION

This case–control study provides many evidences to link primary malignant brain tumors in Kashmiri orchard workers with pesticides, with an OR of >10.00, 95% CI >25–40 and RR of 10.6. Although chemically there appear prominent variations in the serum AChE levels between Kashmiris exposed to pesticides and people from other geographical locations, an OR of >5.00, 95% CI >1–10 and a huge RR of 19.4 predicts decreased levels in cases more frequently. The reason for the altered levels of AChE enzyme in patients may be either different, non-cholinergic mechanism of action of organophosphates, triggered through cAMP; or continuous chronic poisoning, rather than acute, which depresses the AChE levels; or a mixture of different pesticides with different actions on the central nervous system. The causes may also be racial, genetic or immunological. This study lacks the serum AChE levels in hospitalized controls. Clinically, the link between the pesticides and brain cancer appears quite strong and possible but accurate epidemiological studies are yet to document this association. This is in part due to lack of study of action of a single pesticide in an individual case that is exposed to multiple pesticides in one time. However, laboratory and animal studies are in favor of such a link. The familial gliomas are emerging at a high rate in the pesticide workers which is alarming. In the future, studies are needed to accurately localize the link.
